# *Lycoris wulingensis*, a dwarf new species of Amaryllidaceae from Hunan, China

**DOI:** 10.3897/phytokeys.177.62741

**Published:** 2021-04-21

**Authors:** Si-Yu Zhang, Ying Huang, Pei Zhang, Ke-Run Zhu, Yong-Bing Chen, Jian-Wen Shao

**Affiliations:** 1 College of Life Sciences, Anhui Normal University, Wuhu, Anhui 241000, China; 2 Linli Jidong Cement Limited Company, Changde, Hunan 415200, China; 3 Sanjie Nationalized Forest Farm, Chuzhou, Anhui 239400, China; 4 The Key Laboratory of Conservation and Employment of Biological Resources of Anhui, Anhui Normal University, Wuhu, Anhui 241000, China

**Keywords:** Amaryllidaceae, China, *Lycoris wulingensis*, morphology, taxonomy

## Abstract

*Lycoris wulingensis* S.Y. Zhang, a new species from Hunan Province (central South China), is described and illustrated. This new species is a fertile diploid plant and its karyotype is 2n = 22. It is most similar to L. × haywardii in morphology, but the latter is a hybrid species and distributed in East China and the plant is much larger. Amongst the original species, *L. wulingensis* is similar to *L. radiata*, but differs from it in its flowers being rose-red (vs. red) and stamens and tepals are nearly the same length (vs. stamens significantly longer than tepals).

## Introduction

*Lycoris* Herb. (Amaryllidaceae) is a genus distributed only in Asia, including about 24 species, 19 of which are distributed in China, which is the distribution centre of this genus (Hsu et al. 1994; Ji and Meerow 2000; Kim 2004; Hori et al. 2006; Quan et al. 2013; Meng et al. 2018; Lu et al. 2020). Due to the high compatibility amongst most interspecific crosses, hybrids are very common in *Lycoris*. Amongst the 21 karyotype reported species, only seven species are original diploid and the remaining 14 are hybrid species, including allotriploid (such as *L. incarnata*, 2n = 4M+3T+1m+22A = 30), euploid (such as L. × haywardii, 2n = 22A = 22) and aneuploid hybrids (such as L. × albiflora, 2n = 5M+1T+11A = 17) (Kurita 1986; Hsu et al. 1994). The karyotype is an important auxiliary method for identifying *Lycoris* species.

Although there were 43 taxa names of *Lycoris* in the International Plant Name Index (IPNI, https://www.ipni.org/), Hsu et al. (1994) researched and revised the species taxonomy in this genus, based on hybridisation experiments, cytology and morphology and only recognised 20 species and seven varieties. In Korea, Kim revised native *Lycoris* and published two new species, i.e. *L. flavescens* and *L. uydoensis* (Kim 2004). Recently, three new species of *Lycoris* have been discovered and reported in China, namely *L. hunanensis* (Quan et al. 2013), L. × hubeiensis (Meng et al. 2018) and *L. tsinlingensis* (Lu et al. 2020).

During the long-term investigation and collection of Chinese *Lycoris* plant resources over many years, we accidentally discovered this dwarf unique *Lycoris* in the Wuling Mountains area (Hunan Province) in 2016. After four years of observation and cultivation, we confirm that it is a new species and it is described here.

## Materials and methods

To observe and compare morphology characters, about 270 bulbs from nine populations (Table 1) of the putative new species and its relatives (*L. radiata* and L. × haywardii) were collected and brought back for cultivation in August 2015 or 2016. In 2019, the morphological data for bulb diameter, leaf length and width and flower size (tepal length) were measured and recorded from cultivated populations. In 2020, bulb roots were induced by burying in wet sand and the chromosome number was observed using the methods described by Chen and Li (1985). Pollen vitality was tested using the TTC staining method (Oberle and Watson 1953). All statistical analyses were performed in SPSS ver. 19.0.

**Table 1.** The information of sampled populations.

Code		Locations	Altitude
*L. wulingensis*			
	A1	Matouxi Village, Yongding District, Zhangjiajie City, Hunan Province	276 m
	A2	Xiejiapu Village, Shimen County, Changde City, Hunan Province	75 m
	A3	Fawang Village, Taoyuan County, Changde City, Hunan Province	52 m
L. × haywardii			
	B1	Heyi Village, Beilun District, Ningbo City, Zhejiang Province	30 m
	B2	Shanjuan Village, Yixing City, Wuxi City, Jiangsu Province	59 m
	B3	Shanhu Village, Linhai City, Zhejiang Province	18 m
*L. radiata*			
	C1	Luogongpo Village, Wulingyuan District, Zhangjiajie City, Hunan Province	286 m
	C2	Luojiarong Village, Taoyuan County, Changde City, Hunan Province	52 m
	C3	Shanbanqiao Village, Linli County, Changde City, Hunan Province	92 m

## Taxonomic treatment

### Lycoris wulingensis

Taxon classificationPlantaePhyllodocidaNereididae

S.Y. Zhang sp. nov.

1B44D616-36CF-5759-BD45-14786027B365

urn:lsid:ipni.org:names:77216601-1

Figs 1 , 2

#### Type.

China. Hunan, Zhangjiajie County, Wangjiaping Town, Matouxi Village, 29°0'54.7"N, 110°48'3.7"E, under broad-leaved forest, beside the water ditch, 276 m a.s.l., 22 August 2020, *S.Y. Zhang*, *ZSY202008001* (holotype: ANUB; isotypes: PE, KUN).

#### Diagnosis.

Most similar to L. × haywardii, but differs from it by smaller plant and flower sizes (Figs 3, 4, Table 2) and it is restricted to north-western Hunan Province (Fig. 5).

**Table 2.** Comparison the morphology and distribution of *Lycoris wulingensis* and its related species.

Characters	*L. wulingensis*	*L. × haywardii*	*L. radiata*
Leaf	15–27 cm long, 3–5 (mean = 4.0) mm broad	40–55 cm long, 8–12 mm broad	25–45 cm long, 5–10 mm broad
Bulb	2–3 cm in diameter	3.5–5.5 cm in diameter	3–4.5 cm in diameter
Scape	25–30 cm tall, 4–6 mm in diameter	40–50 cm tall, 8–12 mm in diameter	35–40 cm tall, 6–9 mm in diameter
Flower	Flower rose-red, tepals 2.5–2.8 cm long, apex slightly reversed and slightly undulate, stamen 3–3.5 cm long.	Flower rose-red, tepals 4.5–6 cm long, apex slightly reversed and slightly undulate, stamen 6–8 cm long.	Flower red, tepals 3–3.5 cm long, strongly reversed and undulate, stamen 6–8 cm long.
Distribution	North-western Hunan (China)	Southeast Anhui, Southern Jiangsu, Eastern Zhejiang (China)	Southeast Asia; Southwest, South and East China; Japan; South Korea

#### Description.

Perennial herb. Bulbs nearly spherical, 2–3 cm in diameter, covered brown epidermis, with fine lines on the epidermis. Leaves ligulate, often 4–7, blunt apex, appearing in mid-September, 15–27 cm long, 3–5 mm wide; upper surface dark green, mid-vein distinctly pale; bottom surface light green with a raised mid-rib. Inflorescence scapose, 25–30 cm high, green or reddish-brown; 2 spathe bracts, lanceolate, 2–2.5 cm long, 5–7 mm wide, semi-closed to wrap the bud; 3–7 flowers per umbels, pedicels 1.5–2 cm long, diameter 1.5–2 mm; flowers rose-red; perianth lobes oblanceolate, 2.5–2.8 cm long, about 5 mm wide, apex slightly reversed and undulate; floral tubes light red, about 3 mm long. Filaments 3–3.5 cm long, rose-red, slightly longer than tepals, anther yellow, 2–3 mm long; pistil length 4–4.5 cm, middle and lower part diameter 0.8 mm, rose-red, apex diameter about 0.4 mm, dark-red. Ovary 4 mm in diameter, spherical and green. Capsules three-lobed, green or pale when mature; seeds black, spherical, 5–7 mm in diameter.

**Figure 1.**
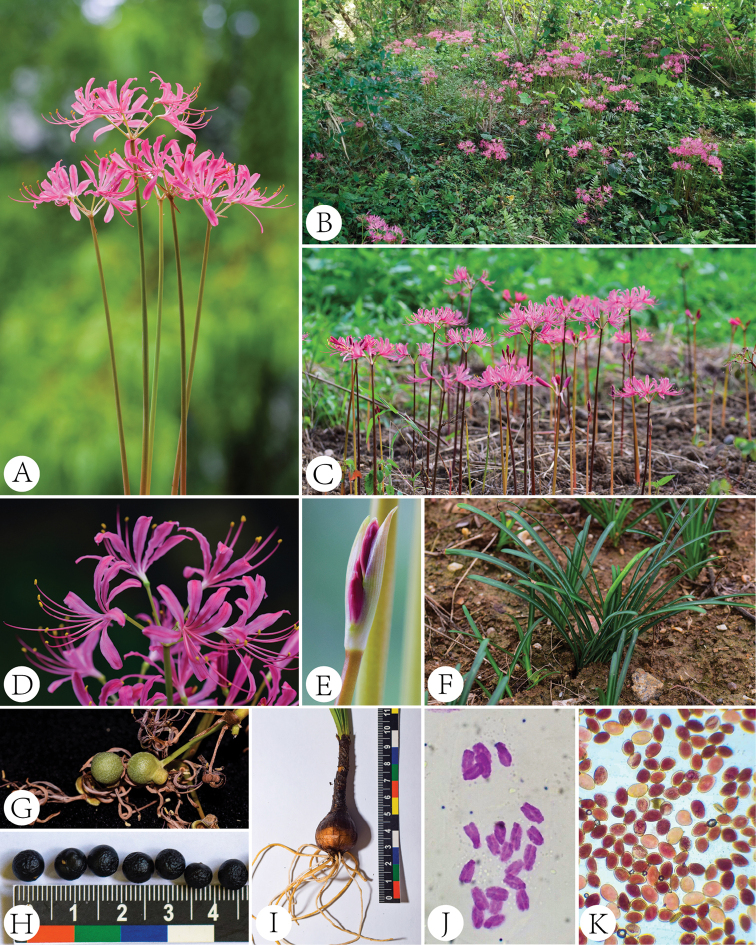
Morphology of *Lycoris wulingensis* S. Y. Zhang, sp. nov. **A** inflorescence **B, C** habitat **D** flower **E** flower bud **F** leaf **G** fruit **H** seeds **I** bulb **J** karyotype (2n = 22) **K** pollen (stained by TTC).

#### Phenology.

Flowering from mid-July to late-August; fruiting in September; and leaves growing in mid-September.

**Figure 2.**
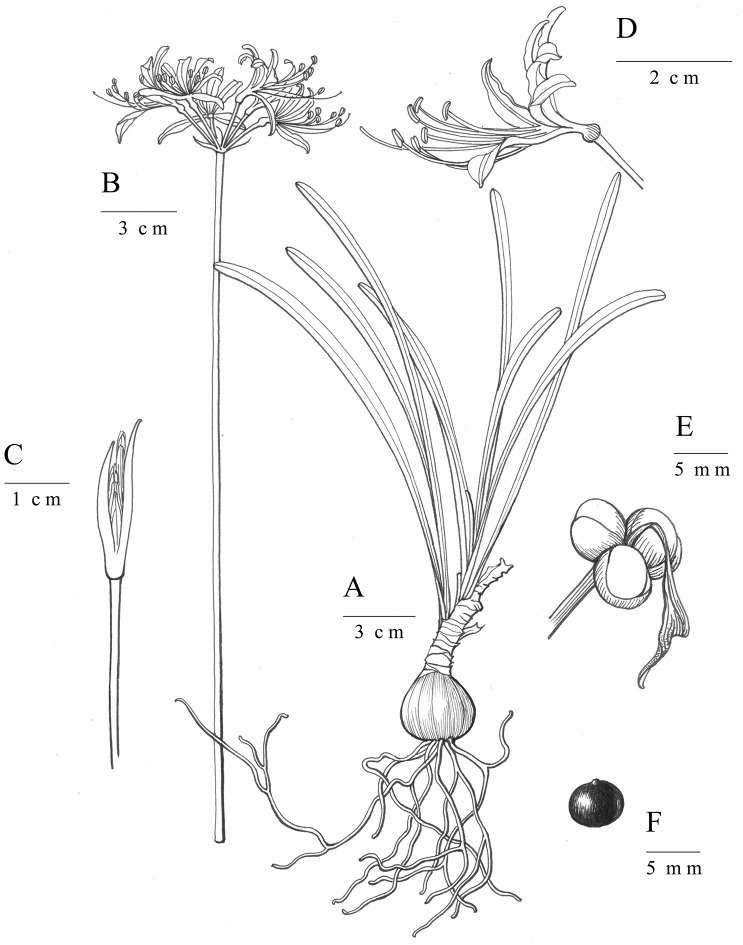
Morphology of *Lycoris wulingensis* S. Y. Zhang, sp. nov. **A** plant **B** inflorescence **C** flower bud **D** flower **E** fruit **F** seed. The picture was drawn by Ling Wang.

#### Distribution.

*Lycoris wulingensis* is distributed in the east of Wuling Mountains and its surrounding areas, such as Cili, Linli, Li, Taoyuan and Yongding Counties (Fig. 5).

#### Habitat.

*Lycoris wulingensis* is partial to grow on the edge of forest roads, farmland or riverside beaches, usually under deciduous trees (such as *Alangium chinense* and *Pterocarya stenoptera*) and accompanied by *Pinellia ternata*, *Commelina communis*, *Arthraxon hispidus* and *Ophiopogon bodinieri* etc. The surrounding residents sometimes cultivate it as an ornamental plant.

#### Vernacular name.

武陵石蒜 [wǔ líng shí suàn].

#### Etymology.

The specific epithet comes from its distribution area of the Wuling Mountains, which is an important biodiversity hotspot in South Central China.

#### Karyotype.

2n = 22 (Fig. 1J).

#### Reproduction.

This species can reproduce asexually by duplication of bulbs (1.5–2 times per year) and can also sexually reproduce through seeds.

#### Conservation status.

Compared with other species in the *Lycoris*, the distribution range of *Lycoris wulingensis* is relatively narrow. Based on preliminary investigation, we found only four large populations and some sporadic distribution points. However, considering the lack of in-depth investigation and also considering that some populations may occur in other similar habitats, we classified its conservation status as Data Deficient (DD), according to the IUCN Red List Criteria (IUCN 2019).

## Discussion

**Figure 3.**
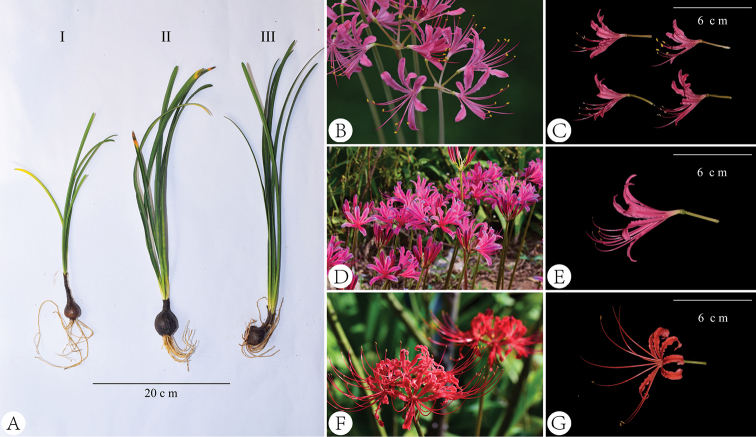
Comparison morphology of *Lycoris wulingensis* and similar species **A** plants (**I**-*L. wulingensis*, **II**-L. × haywardii, **III**-*L. radiata*) **B–G** flowers (**B, C***L. wulingensis***D, E**L. × haywardii**F, G***L. radiata*).

**Figure 4.**
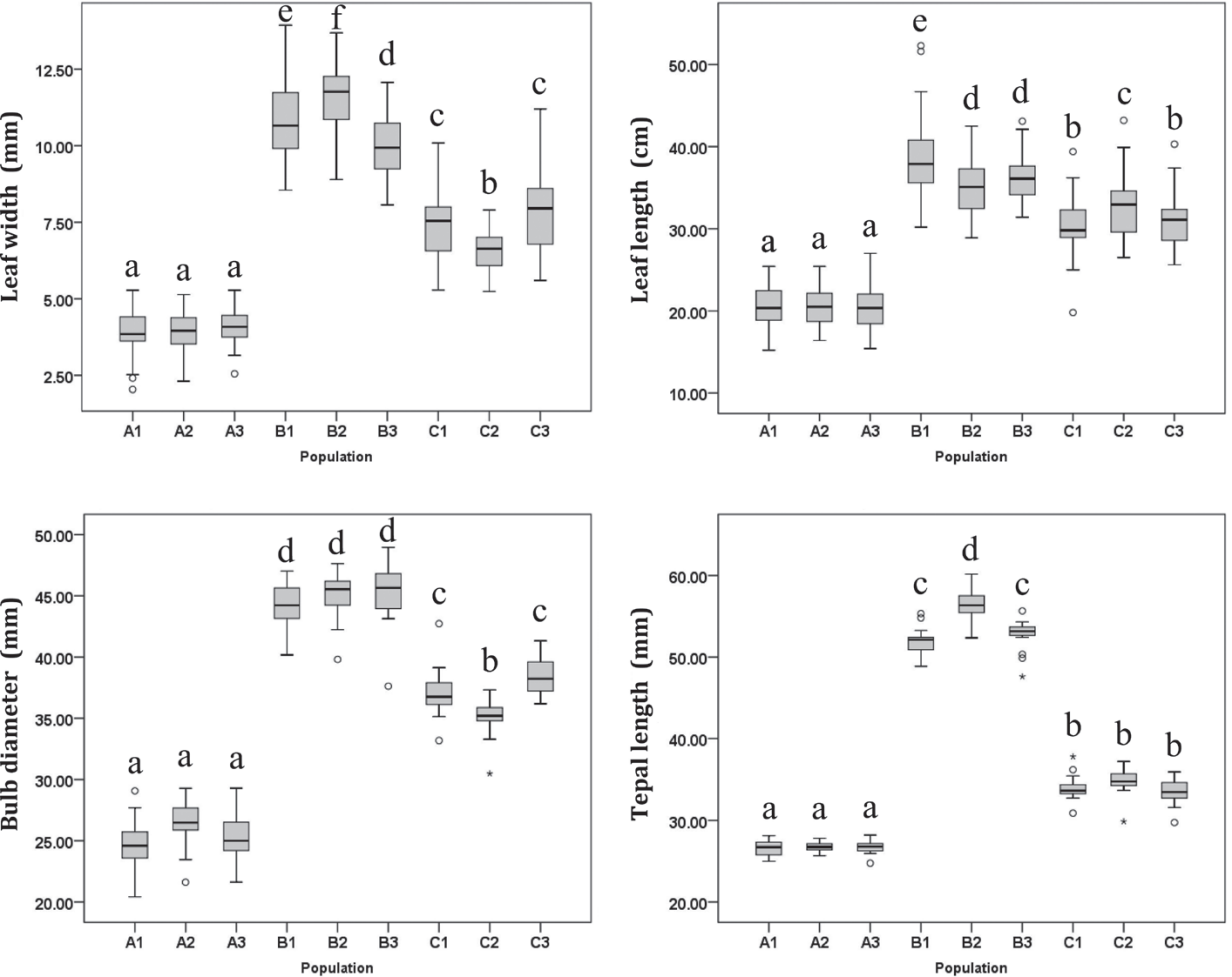
Comparison and variation of leaf width and length, bulb diameter and tepal length (flower size) of *L. wulingensis* (A1–A3), L. × haywardii (B1–B3) and *L. radiata* (C1–C3). In the boxplot, the horizontal line shows the median, the bottom and top of the box show the first and third quartiles. Boxplot marked with different letters differ significantly (post hoc test, P < 0.05).

After four years of cultivation and observation, we found that the plant size of *Lycoris wulingensis* was consistently small (Figs 3, 4). Amongst different wild populations and even under different cultivation conditions, its leaf length was always less than 27 cm, bulb diameter was about 2–3 cm and flower tepal length was less than 3 cm (Figs 3, 4). To our knowledge, the body size of this new species is the smallest in *Lycoris* (Hsu et al. 1994; Ji and Meerow 2000). The flowers of *L. wulingensis* are rose-red and their filaments are nearly equal to tepals in length, which is most similar to L. × haywardii. However, previous hybridisation experiments, molecular studies and field investigations have shown that L. × haywardii is a hybrid species which is only distributed in eastern China (the overlapping area of its two parents, *L. radiata* and *L. sprengeri*) (Hsu et al. 1994; Shi et al. 2006), while *L. wulingensis* is now known to be distributed in the east of Wuling Mountains and its surrounding areas in north-western Hunan Province, central South China. In addition, possibly due to the scape of *L. sprengeri* (one parent) being tall and strong, the scape of L. × haywardii is stronger and longer than that of *L. radiata* and *L. wulingensis* (Figs 3, 4) and the flower of L. × haywardii is about twice the size of *L. wulingensis* (Figs 3, 4). In terms of leaf morphology, *L. wulingensis* and *L. radiata* both have narrow dark green leaves with a clear white band in the centre. However, there are many obvious differences between these two species in flower characters, such as the colour of the former being rose-red and the stamen length is about equal to the petal length, while the latter’s are red and stamens are about twice as long as the tepals. Thus, it is easy to distinguish *L. wulingensis* from its related species by plant size, floral characters and distribution range (Table 2).

**Figure 5.**
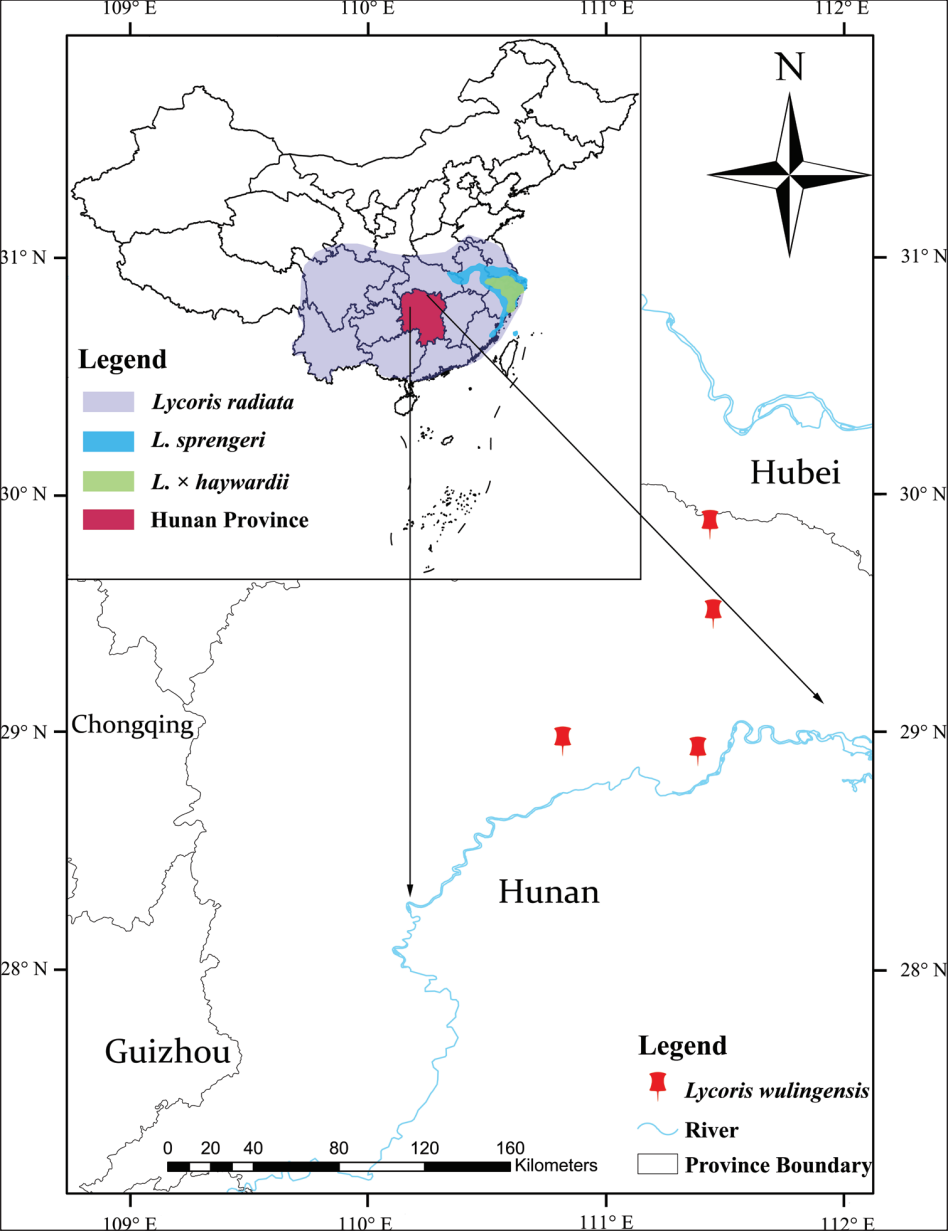
Distribution map of *Lycoris wulingensis* S.Y. Zhang and its related species.

Initially, we speculated that *L. wulingensis* was a haploid type of *L. radiata* and its dwarfism characters were possibly induced by the loss of half of its chromosomes. However, its chromosome number is 2n = 22 (Fig. 1J), which is similar to the original species of *L. radiata*, *L. sprengeri* and *L. sanguinea* (Hsu et al. 1994; Hori et al. 2006). TTC staining showed that the vitality of its pollen was normal (Fig. 1K). Furthermore, *L. wulingensis* can sexually produce offspring by seeds in wild habitats and under cultivated conditions and the seeds can also germinate and develop into seedlings. Therefore, we suppose that *L. wulingensis* is likely to be an original diploid species, which possibly has high value in terms of horticultural breeding.

## Supplementary Material

XML Treatment for Lycoris wulingensis
